# Sexual assault by a gynecologist with two frontal lobe meningiomas: implications and challenges for forensic psychiatric assessment: a case report

**DOI:** 10.3389/fpsyt.2026.1692213

**Published:** 2026-03-31

**Authors:** Carla Occhipinti, Marco Di Paolo, Ilaria Zampieri, Pietro Pietrini

**Affiliations:** 1 Institute of Legal Medicine, Department of Surgical Pathology, Medical, Molecular and Critical Area, University of Pisa, Pisa, Italy; 2 Molecular Mind Lab, IMT School for Advanced Studies Lucca, Lucca, Italy

**Keywords:** Behavioral disinhibition, criminal responsibility, forensic psychiatry, frontal meningioma, imputability

## Abstract

Brain diseases may determine the development of focal neurological deficits and behavioral anomalies. In some cases, behavioral alterations may lead to illicit demeanours which, in turn, may have potential serious legal consequences as well. We report the case of a male gynecologist in his seventies sued by a young female patient for sexual violence. The man had no significant premorbid medical history and was a respected medical doctor with several decades of professional activity. Months after the sexual violence episode, he began to manifest symptoms indicative of cognitive impairment, which led to a neurological examination including a Magnetic Resonance Imaging (MRI) brain scan exam. Brain MRI revealed two frontal meningiomas with mass effects and vasogenic oedema on the surrounding brain parenchyma, for which the patient underwent a neurosurgical operation. Following surgery, partial improvement in selected cognitive and behavioral domains was observed, suggesting that the frontal lesions may have contributed significantly, though not exclusively, to the clinical picture. In light of this evidence, the question arises whether and to what extent the mental capacity of the subject at the time of sexual offense could have been compromised by the presence and location of the tumors, that is, as a potential “precipitating factor” impinging on a brain with underlying neurodegenerative and/or vascular lesions.

## Introduction

It is well known that a variety of brain disorders may also cause cognitive impairment, as well as personality and behavioral abnormalities. These alterations may occur following structural and/or metabolic changes in regions of the brain – including, in particular, frontal lobes, amygdala, and hypothalamus - that are involved in executive functions, self-awareness, emotional processing, language, and control of aggressive, social and sexual behavior (1, 2).

Among others, widely known is the case of Phineas Gage, a young American railroad worker, who survived an accident in which an iron rod was driven through his head, destroying much of his left frontal lobe. In the period following the accident, Gage manifested a disruption of his previous behavior, becoming easily irritable, short-tempered, anaffective, violent, and disrespectful. He also began to use obscene language and lost decision-making and organizational skills (3).

While neurological and cognitive consequences may become rapidly evident following acute traumatic or vascular events (4), more subtle personality and behavioral changes may unveil over time in the course of several neurodegenerative disorders, including frontotemporal dementia, Alzheimer’s disease, Parkinson’s disease (5), vascular dementia (6), Huntington’s chorea (7), as well as in association with epilepsy (8) and multiple sclerosis (9). Furthermore, neoplastic formations (10) or invasive brain surgery (11) also may cause dramatic personality and behavioral alterations.

In some cases, behavioral changes represent the very first clinical manifestation of the disease, in the absence of any other detectable and/or clear cognitive or neurological symptom or sign, and may also result in illicit and criminal behaviors (12). Although such cases are not very frequent, they still pose a major challenge because of the social and legal consequences for the patients and their families, as well as for the whole community.

Here, we report the case of a male gynecologist in his seventies who was sued by a patient for sexual violence during a medical examination. Shortly after that episode, the doctor began to show cognitive impairment and was eventually diagnosed with multiple meningiomas in the anterior portion of the left cerebral hemisphere.

Informed consent for publication of this case report was obtained from the defendant and his family. The manuscript focuses exclusively on the clinical and forensic aspects of the defendant’s neurological condition.

The victim is in no way identifiable from the information provided in this manuscript. No personal, demographic, geographic, or contextual details that could even remotely lead to the victim identification are included. All the information has been carefully anonymized and limited to what was considered to be strictly necessary for scientific and clinical purposes.

## Case report

A male gynecologist in his seventies was reported for sexual assault by an adult patient. The woman had contacted the doctor, in October 2021, because of problems with her menstrual cycle. She reported that during the medical examination, the doctor carried out some clinical maneuvers that were not supposed to be part of a routine gynecological examination and were clearly inappropriate. Specifically, the doctor touched the woman’s intimate parts and went on to stimulate them for several seconds, using his bare hands.

Moreover, upon completion of the gynecological examination, the doctor reportedly began to inquire about the patient’s sexual habits, asking questions that appeared to be completely unrelated to the reasons for the visit and well outside of any medical context. The patient referred that throughout the examination, the doctor behaved as if he were oblivious to the inappropriateness of his doing. Shortly after these events, the doctor ceased his professional activity.

He had been married with the same woman for several decades and the couple had three grown-up children. His family and friends depicted him as a cheerful and joyful person, passionate about reading and keen on learning languages. He was a respected professional who had worked in a public hospital until his mandatory retirement and had continued to work as a private gynecologist afterwards. He had no previous history of malpractice or legal issues.

According to his wife and his daughter, who were physicians as well, the doctor had never shown any significant neurological, psychiatric, or psychological problems. He only suffered from hypertension, which was under pharmacological control, mild diabetes and had recently developed a benign prostate enlargement under hormonal treatment. Retrospectively, both his wife and daughter reported that over the last couple of years prior to the criminal facts (around 2019-2020), the doctor had begun to show emotional liability, characterized by increased irritability and a progressive mood deflection that eventually evolved into depressive symptoms. At the time, his family interpreted these changes as a normal emotional reaction to retirement and the consequent reduction of daily professional and social engagement. Over the time, these alterations became more and more frequent and severe and were then followed by language and memory deficits and motor slowdown. Following the criminal allegation in 2021, the overall cognitive condition became more significantly manifest and was accompanied by depressive symptomatology in reaction to the legal problem. However, a formal neurological consultation was sought only in October 2023, when the worsening of the clinical picture became definitely very pronounced.

Neurological examination revealed disorganized speech and ideational apraxia. The doctor appeared to have absolutely no insight into his condition and his difficulties. Administration of the Mini-Mental State Examination (MMSE (13)), revealed a score of 15/30 (13.3/30 after correction for age and education). Neurological examination was otherwise negative. The neurologist suspected a potential dementia process and recommended follow-up with a brain MRI scan examination.

**Figure 1:**
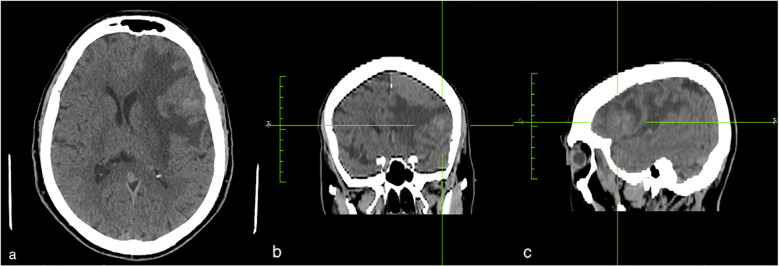
MRI of the brain revealed a meningioma located in the antero-inferior region of the frontal operculum. It measured 21x24x25 mm and was associated with vasogenic edema a transfalcine herniation. The figures show the axial **(a)**, coronal **(b)**, and sagittal **(c)** projection of the lesion.

**Figure 2:**
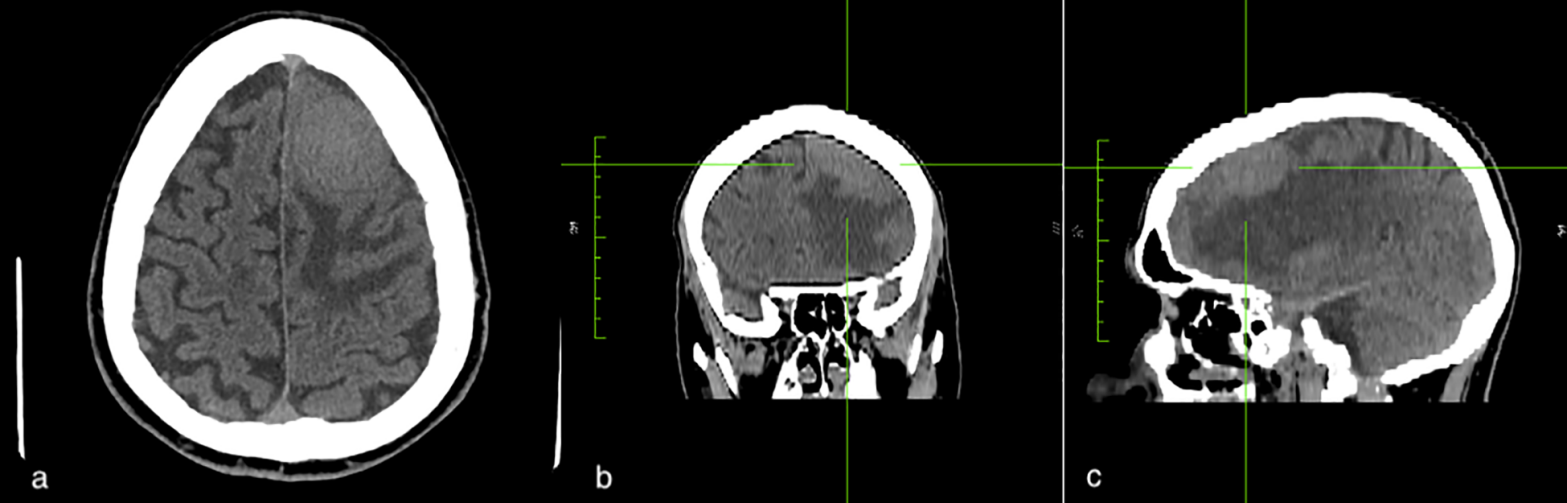
The second meningioma measured 32x34x35 mm and was located at the anterior vertex of the frontal lobe. The figures show the axial **(a)**, coronal **(b)**, and sagittal **(c)** projection of the lesion.

MRI scans (performed in October 2023) revealed the presence of two meningiomas, both located on the left side of the brain. The smaller one measured 21 x 22 x 25 mm and was situated in the antero-inferior region of the frontal operculum. This meningioma resulted in vasogenic oedema of the surrounding parenchyma, a right displacement of median structures with transfalcine herniation, and partial obliteration of the ipsilateral ventricle (**Figure 1**). The second meningioma measured 32 x 34 x 35 mm, was located at the anterior vertex of the frontal lobe and was also accompanied by signs of mass effect (**Figure 2**). Of note, the diagnosis of the frontal meningiomas occurred approximately two years after the criminal allegation (October 2021) and several months before the court-appointed forensic neurological evaluation (June 2024). The neuroimaging findings were detected during a routine MRI scan prescribed by a neurologist as a part of the patient clinical evaluation. Thus, the MRI examination was not prompted by a judicial request.

The patient thus underwent surgery for removal of the two meningiomas in November 2023.

While surgery itself was uneventful, the post-operative course was complicated by an accidental fall of the patient with head trauma and development of extradural hematoma which required a second emergency neurosurgical intervention. Furthermore, during his long hospitalization (over 40 days), the patient contracted Covid disease, developed urinary tract infections with urine culture positive to *Staphylococcus Epidermis*. and *Escherichia Coli* and deep vein thrombosis in the lower legs.

The patient eventually recovered from all the above medical complications and started a rehabilitation program, between December 2023 and February 2024, that allowed him to partially overcome some of the motor and linguistic deficits. Interestingly, after the removal of the meningioma, the patient appeared to have acquired insight into his pathological condition.

A control MRI scan, performed on January 30, 2024, showed a wide gliotic area within the left frontal region with hemosiderin deposits in the inferior cortical gyrus, beside a residual centimetric hemorragic focus in the left inferior frontal cortex (tardive sub-acute hematoma) and post-ischemic gliosis in the right dorsal cerebellar hemisphere. Moreover, multiple gliotic areas within the supratentorial white matter within the corone radiate, semioval centers and in the bilateral subcortical fronto-insular region and in the middle third of the corpus callosum and two small meningiomas (about 7 mm each) within the bilateral superior posterior parietal region with no significant effect on the underlying cerebral cortex were also detected.

At the time of the forensic evaluation by the expert neurologist appointed by the Judge as a preliminary step to the trial (June 2024), the patient was alert though sleepy, relatively confused, bradykinetic, poorly oriented in time, space and toward persons and the situation. He was not able to adequately comprehend the reason and the nature of the visit. He was wheel-chaired and was able to stand only with help; he could walk only for a few steps by using a rollator (wheeled walker) and with the help of a third person. His gait was atassic. He showed a frequent spontaneous spastic crying and laughing. Though formally cooperative during examination, his behavior and language were inappropriate. Mood was flattened. Speech was reduced and not fluent, with some dysarthria. Lexical, grammatical and syntactical elements were altered as compared to his age and education levels. Thought content was incoherent and illogic. No hallucinations or psychotic phenomena were appreciated. He showed many pathological reflexes, including the palmo-mental and the forced grasp reflexes and had no habituation on the glabellar tap sign.

Because of the severity of the cognitive and motor deficits, no formal neuropsychological test could be performed.

The expert concluded that the patient was affected by severe dementia and lacked the capacity to stand trial.

**Figure 3:**
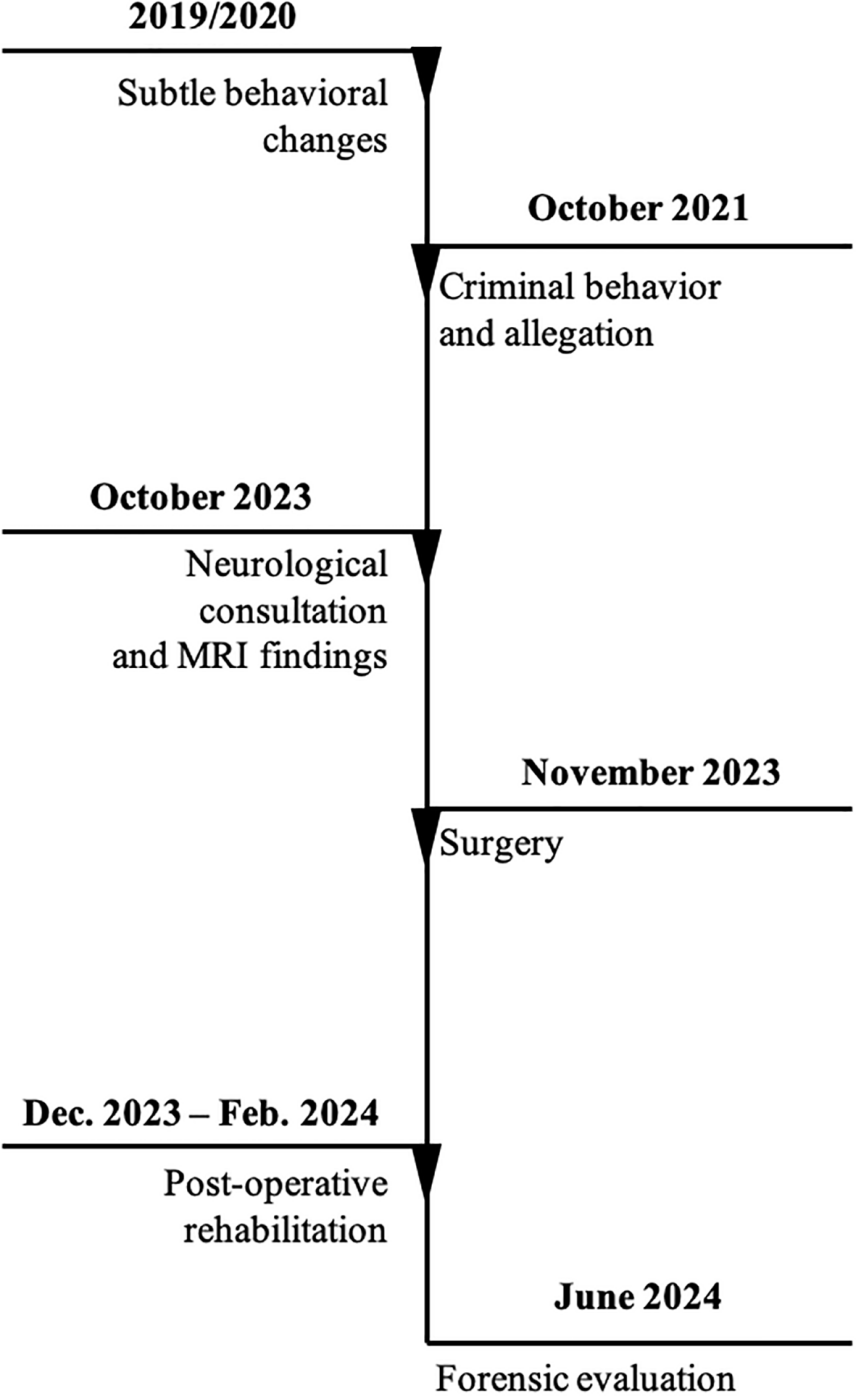
Timeline of the main clinical and legal events.

A timeline of the main clinical and legal events has been reported, summarizing (**Figure 3**): subtle behavioral changes (around 2019/2020), allegation (October 2021), neurological consultation (October 2023), MRI findings (October 2023), brain surgery (November 2023) and post-surgery complications, post-operative rehabilitation (December 2023- February 2024), forensic evaluation (June 2024).

## Discussion

Intracranial tumors determine a variety of neurological manifestations, including behavioral alterations and personality changes. Symptoms and signs depend on the position and volume of the tumor, as well as on the effects caused by the growing mass on the surrounding parenchyma. Therefore, tumors located within the occipital lobes, often cause contralateral hemianopia as an onset sign; tumors involving the parietal lobes typically occur with sensory loss, spatial disorientation, and receptive aphasia (14). Finally, tumors located within the frontal and temporal lobes, are associated with generalized or focal seizures, expressive aphasia, dementia, gait disturbances, personality changes, behavioral alterations (1), including sexual disinhibition (10) (15),, release of uncontrolled aggressiveness (2), and are typically accompanied by lack of insight into one’s own impairment (16).

Indeed, the frontal cortical areas are crucial for higher cognitive functions, including the ability to distinguish right from wrong, abstract thinking, decision-making processes, as well as behavioral control (2) and awareness (17).

While personality and behavioral alterations are the earliest and most common features of disorders that affect the frontal cortical areas, in the event of slow-growing processes, the course of the disease is usually subtle so that symptoms and signs may go undetected – or underestimated - for a long time, until they give rise to a sudden dramatic event. Depending on the context, such disruptive manifestations may have also legal consequences, as it happened in the case reported here.

Meningiomas are benign tumors of the meninges. Because they are usually slow-growing, as mentioned before, most meningiomas remain clinically silent for a long time, with symptoms and signs that occur gradually, and their diagnosis is often delayed (18). This is indeed the case especially for meningiomas located in the anterior frontal regions, for which behavioral and personality alterations, including psychiatric symptoms, may represent the first and only manifestation of the disease (19).

In the case reported here, several aspects of the symptom progression are anatomically coherent with the location of the two frontal lesions and their associated mass effect. The meningioma located in the antero-inferior region of the frontal operculum may plausibly relate to behavioral disinhibition, including abnormal sexual conduct, whereas the lesion at the anterior vertex of the frontal lobe may account for progressive executive dysfunction, speech disturbances, psychomotor slowing, and impaired insight.

Nevertheless, alternative or concomitant diagnoses, including frontotemporal dementia, vascular dementia, stroke or metabolic encephalopathies should have been considered. In fact, during the neurological consultation in 2023, the examining neurologist raised the suspicion of an underlying dementia process. The diagnostic picture, indeed, cannot be considered entirely univocal. The patient was in his seventies, an age group in which neurodegenerative and vascular cognitive disorders become highly prevalent. Moreover, the markedly reduced MMSE score (13.3/30 after correction for age and education) appears relatively severe when interpreted solely in relation to the size and location of the lesions. While frontal meningiomas are well known to cause, by themselves, prominent executive dysfunction, behavioral disinhibition and impaired insight, such a low global cognitive score may suggest either extensive functional disruption secondary to mass effect or the possible coexistence of an additional neurodegenerative or vascular process. Indeed, recent literature has highlighted that cognitive impairment is highly prevalent among older defendants referred for forensic psychiatric evaluation. In a descriptive study of individuals aged 60 and older evaluated within a forensic setting, approximately 42% were diagnosed with a cognitive disorder and nearly one third were deemed incompetent to stand trial. Notably, sexual charges represented one of the most frequent categories of alleged offenses in this population (39%). These findings underscore the clinical and legal relevance of systematically assessing cognitive status in elderly defendants, particularly in cases involving late-onset or atypical sexual behavior or abuse (20).

However, it should be emphasized that, in the present case, the brain MRI performed in October 2023 — which led to the diagnosis of the two frontal meningiomas — did not describe radiological signs suggestive of overt diffuse neurodegenerative pathology or significant microvascular disease or any other lesion beyond the two expansive lesions and their associated mass effect. Thus, at that stage, the brain structural abnormalities appeared confined to the neoplastic lesions. Nevertheless, the absence of clear degenerative markers on routine structural imaging does not entirely exclude the possibility of an early or clinically evolving neurodegenerative condition, particularly in elderly individuals. Thus, a possibility exists that the two frontal meningiomas may have acted as a decompensating/precipitating factor of an underlying vulnerable brain condition, rather than having an exclusive causative role.

The temporal relationship between the onset of subtle behavioral and emotional changes, the later (severe) cognitive deficits and the discovery of two neoplastic lesions within the frontal lobes on one hand and the illicit sexual conduct on the other hand, raises the question of whether and to what extent the doctor was capable of understanding the nature of his actions and/or to properly behave at the time of the alleged crime. As it is well known, the penal system requires that an individual have both the ability to understand and the ability to will in order to be considered responsible for his or her actions (art. 85 of the Italian Penal Code). Furthermore, an individual must possess sufficiently preserved cognitive abilities in order to be able to stand in court at the time of the trial.

According to the Italian legal system, the assessment of criminal responsibility distinguishes between total and partial mental incapacity. In cases of total incapacity, the defendant is considered not criminally liable, whereas partial incapacity entails a reduction of the sentence and may also lead to the application of security measures. This framework reflects the legislator’s intent to proportion criminal liability to the degree of mental impairment, underscoring the pivotal role of forensic psychiatric evaluation in determining imputability and guiding judicial decisions (21).

In this specific case, in accordance with the principles set forth by statutory law and the jurisprudential doctrine within the Italian legal system, several factors concur to support the hypothesis of significantly compromised mental capacity at the time of the alleged conduct. As a matter of fact, the illicit conduct appears to be in sharp contrast with a four-decade-long career as an estimated medical specialist and a well-respected community member, indicating a sort of “behavioral fracture” between the past and the present event. Furthermore, according to the same victim’s report, the doctor appeared to be completely unaware of the social disvalue and the wrongfulness of his doing. This appears to be in line with his complete lack of insight, as he had no awareness of any cognitive difficulties in contrast to the remarkable deficit revealed by the neurological assessment. The severely impaired MMSE score and the presence of frontal release signs further corroborated the existence of substantial frontal dysfunction. The cognitive and behavioral improvement, though partial, observed following surgical removal of the meningiomas —including recovery of some speech abilities, recover of psychomotor slowing and acquisition of insight into his clinical condition— does support a contributory role of the frontal lesions to the neuropsychiatric deterioration. Notably, this regained insight referred to awareness of his pathological state, as documented in the clinical records, and clearly did not extend to retrospective awareness of the inappropriateness or wrongfulness of the alleged conduct.

Despite the fact that the two meningiomas were removed successfully, the patient eventually progressed to a condition of severe cognitive deterioration and neurological impairment. The overall neurological and cognitive condition at the time of the forensic evaluation appears to be the expression of concurrent events, specifically, the sequelae of the accidental head trauma with intracranial hemorrhage and subdural hematoma and the underlying microvascular sufferance.

However, the assessment of the effective role of the underlying pathological condition in the genesis and dynamics of the illicit act may be challenging, as the mere neuroradiological evidence of a brain structural and/or functional alteration may not be thought to be sufficient to prove a causal link with the abnormal behavior, especially when the individual overall retains a general condition of relative well-being and functioning in daily activities (22). Furthermore, typically the expert evaluation ordered by the Judge follows by at least several months the time of the event, making it more difficult for the specialists to determine the real patient conditions “at the time of the facts”, as it is required by current Italian law.

These considerations highlight the importance of timely medical and neuropsychiatric evaluation in elderly defendants, particularly when late-onset behavioral changes, disinhibition or atypical sexual conduct emerge in individuals without any prior history of misconduct and represent a sort of “behavioral fracture” with personal history. Early multidisciplinary assessment —including neurological examination, comprehensive neuropsychological testing and brain imaging— may facilitate accurate diagnosis, allow prompt therapeutic interventions, and provide a more reliable documentation of the individual’s mental state closer to the time of the alleged offense. Such an approach may reduce retrospective uncertainty and enhance both the clinical management and fairness of judicial proceedings.

Finally, the fact that, unlike all the other medical disciplines, (forensic) psychiatry still suffers from the lack of accepted and validated objective measures, most often makes forensic psychiatric assessments to be highly subjective and controversial, with a dramatically different impact on the defendant’s legal course.

We propose that a multidisciplinary integrated approach that combines medical history, clinical evaluation and neuroradiological and neuroscientific exams may reduce subjective variance, thus enabling forensic psychiatry to assume the same objective status as the remaining medical disciplines (15, 23, 24). It must be acknowledged that, at present, such an integrated approach that combines clinical, neuropsychological, and neuroradiological information can only be applied in a limited subset of cases—namely, those in which a clear structural or organic brain pathology is identifiable, as in the present report. Nonetheless, the broader goal of forensic neuroscience is to develop objective and quantifiable measures of brain structure and function that could, in the future, contribute to a more reliable and evidence-based assessment of criminal responsibility.

## Conclusions

A variety of brain disorders, including brain tumors, are characterized by impaired cognitive functions and behavioral changes. In some cases, behavioral abnormalities represent the first and most prominent clinical manifestations of the disease; at times, they may also give rise to illicit and criminal conducts.

Here we reported a case of a medical doctor who showed inappropriate and illicit sexual behavior toward a young patient of his and was shortly afterwards found to have a severe cognitive impairment with no insight into his pathological condition. Brain MRI scans revealed two large meningiomas with mass effects on the frontal lobes. After their surgical removal, the patient partially recovered from his cognitive impairment and gained insight into his medical state.

From a forensic point of view, in cases like the one presented here, it is not always easy to assess and quantify the mental capacity of the subjects at the time of the event and, consequently, their imputability. The mere detection of the tumors, documented by brain imaging studies, may not be sufficient to settle the issue. This is because neuroimaging and functional investigations alone do not have yet a sufficient evidentiary weight to be considered more than a mere piece of documentation. However, when evaluating the causative link between the pathological condition and the criminal act – as it is required by law – it is crucial to take into consideration alternative hypothesis as well. In the specific case, if one intended to dispute the causal role of the two brain meningiomas on the disinhibited and illicit behavior enacted by the defendant, alternative explanations should be carefully considered and critically evaluated for an act that was in sharp contrast with a four decades of impeccable professional activity and appeared to be carried out with no awareness of its inappropriateness on the doctor’s side, as affirmed by the victim herself.

At the moment, a multidisciplinary integrated approach that combines medical history, clinical evaluation and neuroradiological and neuroscientific exams appears to be the optimal way to achieve a valid interpretation and reduce the subjective variance usually observed within the context of forensic assessments in these cases (15, 23, 24). Although such an integrated clinical and neuroradiological approach is currently feasible mainly in cases with identifiable organic pathology, it outlines the direction forensic neuroscience aims to pursue the development of objective, measurable indicators of brain function that may, in the future, contribute to a more consistent and evidence-based assessment of criminal responsibility.

## Data Availability

The original contributions presented in the study are included in the article/supplementary material. Further inquiries can be directed to the corresponding author.
